# Delocalized Electric Field Enhancement through Near-Infrared Quasi-BIC Modes in a Hollow Cuboid Metasurface

**DOI:** 10.3390/nano13202771

**Published:** 2023-10-16

**Authors:** José Francisco Algorri, Victor Dmitriev, José Miguel López-Higuera, Dimitrios C. Zografopoulos

**Affiliations:** 1 Photonics Engineering Group, University of Cantabria, 39005 Santander, Spain; lopezhiguera@unican.es; 2 CIBER de Bioingeniería, Biomateriales y Nanomedicina, Instituto de Salud Carlos III, 28029 Madrid, Spain; 3 Instituto de Investigación Sanitaria Valdecilla (IDIVAL), 39011 Santander, Spain; 4 Electrical Engineering Department, Federal University of Para, Agencia UFPA, P.O. Box 8619, Belem 66075-900, PA, Brazil; victor@ufpa.br; 5 Consiglio Nazionale delle Ricerche, Istituto per la Microelettronica e Microsistemi, 00133 Rome, Italy; dimitrios.zografopoulos@artov.imm.cnr.it

**Keywords:** dielectric metasurfaces, bound states in the continuum, sensing devices, spectroscopy

## Abstract

The two main problems of dielectric metasurfaces for sensing and spectroscopy based on electromagnetic field enhancement are that resonances are mainly localized inside the resonator volume and that experimental Q-factors are very limited. To address these issues, a novel dielectric metasurface supporting delocalized modes based on quasi-bound states in the continuum (quasi-BICs) is proposed and theoretically demonstrated. The metasurface comprises a periodic array of silicon hollow nanocuboids patterned on a glass substrate. The resonances stem from the excitation of symmetry-protected quasi-BIC modes, which are accessed by perturbing the arrangement of the nanocuboid holes. Thanks to the variation of the unit cell with a cluster of four hollow nanocuboids, polarization-insensitive, delocalized modes with ultra-high Q-factor are produced. In addition, the demonstrated electric field enhancements are very high ($10^{3}$–$10^{4}$). This work opens new research avenues in optical sensing and advanced spectroscopy, e.g., surface-enhanced Raman spectroscopy.

## 1. Introduction

Plasmonic materials, specifically gold and silver, have unique properties for optical sensing. The free electrons of these materials can be coupled with incident light to produce collective oscillations in the form of surface plasmon waves. In the case of optical sensing, different resonant phenomena can be exploited. For example, localized surface plasmon resonances (LSPRs) [1] based on the collective oscillation of electrons in individual nanoparticles allow for very high electric field confinement and significant enhancement. The resonance depends on the shape and size of the nanoparticle and the surrounding dielectric medium. Several examples exist in the literature, with maximum sensitivities around $10^{2}$ nm/RIU and a sensing distance of 10 nm [1]. Another interesting phenomenon is surface plasmon resonances (SPRs) [2]. In this case, the resonance occurs at the interface of a metallic and dielectric material. The surface plasmon wave produces an evanescent field that is strongly dependent on the refractive index of the dielectric material, making it very sensitive to variations of the same. This type of sensor achieves maximum sensitivities around $10^{6}$ nm/RIU and a sensing distance of 1 μm [1]. For this reason, it has been used in numerous optical sensing devices [3]. By functionalizing the metal layer, plasmonic sensors even allow for the detection of single molecules [4]. Finally, another interesting phenomenon is the surface lattice resonances (SLRs) produced in arrays of nanoparticles [5]. These occur when the light scattered by each nanoparticle within an array combines to produce a collective oscillation across the entire array [6]. These collective resonances are spectrally close to the position of the Rayleigh anomaly, where diffraction orders emerge. SLRs produce distinct spectral features, creating narrow resonances in the array’s optical response. SLRs exhibit substantial spatial coherence, extending over areas significantly larger than the light wavelength. For these reasons, it has been proposed for spatially extended refractometric sensing with sensitivities around 100–450 nm/RIU [7].

However, while plasmonic structures have attracted significant interest due to their unique optical properties, they come with a number of drawbacks. They experience ohmic losses that can significantly reduce the system’s efficiency and limit its performance, mainly by inducing significant resonance broadening [8]. Moreover, thermal issues can lead to material degradation or failure, especially when using high-power light sources [9]. A promising alternative lies in the utilization of dielectric materials. Specifically, the optical response of dielectric metasurfaces (MS) depends on Mie resonances, which significantly enhance light’s electric and magnetic field components [10]. Moreover, they facilitate intricate wavefront control, including amplitude, phase, dispersion, and light polarization modulation [11]. Nevertheless, it is important to note that a certain degree of light leakage is inherent to these systems, which limits optimal light confinement and enhancement. One phenomenon that overpasses these issues is bound states in the continuum (BICs) [12]. In the case of photonics, these are a unique class of optical modes localized within a photonic structure and are notable for their immunity to radiative losses [13].

Metasurfaces have recently been identified as a viable platform for engineering BICs. Within MS, several symmetry-protected BICs exist as eigenmodes situated above the light cone. They possess different symmetries than the incoming wave, making them not excitable within the continuum of radiation modes [13]. However, introducing a perturbation to these pure BICs can transform them into quasi-BICs. These quasi-BICs have a finite lifetime, narrow linewidth, and a high radiative Q-factor, making them a topic of intense investigation in the field of dielectric MS with symmetry breaking. This investigation typically involves introducing geometrical asymmetry or using anisotropic materials [14]. The degree of asymmetry influences the strength of electromagnetic coupling between the incoming radiation field and the polarization currents inside the particles [15].

Although in most cases the resonant near-field is mainly confined to the interior of the high-index subwavelength scatterers, where they provide limited light–matter interaction with the surrounding material, it remains feasible to achieve interaction at the interface, allowing for biosensing and integration with 2D materials [16] as well as in slotted regions [17,18]. Furthermore, it has been recently reported that asymmetric dielectric particles arranged in clusters can be used to achieve greater flexibility in obtaining the desired near-field configuration [19] and enhancing the sensing capabilities for refractometry [20]. The operation principle in the latter case is based on plasmon-like surface waves, which are delocalized over a wide area to provide a large interaction volume with the matter but are excited in an all-dielectric structure. The current work investigates a novel dielectric metasurface supporting delocalized quasi-BIC modes. The metasurface comprises clusters of four meta-atoms of silicon hollow nanocuboids patterned on a glass substrate, and is specifically designed to yield ultra-high Q-factor delocalized resonances in the near-infrared telecom spectrum. In previous works, this structure has been demonstrated for toroidal responses [21,22]; the present cluster configuration exhibits enhanced features different from the standpoint of fundamental physics. Interestingly, the electric field is mainly located outside the resonator volume, suggesting its potential in applications where external field interaction is crucial.

## 2. Design and Operation Principle

**Figure 1:**
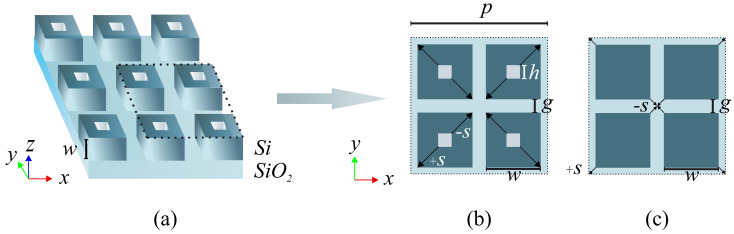
(**a**) Schematic depiction of the silicon MS under study. The square lattice has a pitch of *p*, while the silicon nanocuboid has a volume of $w^{3}$. The MS is patterned atop a silica substrate. The layer on top is considered as a semi-infinite medium with a refractive index of $n_{a}$. (**b**) Unit cell comprising a cluster of four nanocuboids with holes shifted outwardly and inwards in the cluster diagonal from the nanocuboid centres by an offset of *s*. (**c**) Unit cell composed of solid nanocuboids whose center is shifted along the diagonals of the cluster.

The structure depicted in Figure 1a represents the investigated MS consisting of a periodic array of hollow nanocuboids. These hollow nanocuboids are etched into a thin silicon layer (characterized by an index of $n_{\mathrm{Si}}=3.45$ at 1.55 µm [23]) on an SiO${}_{2}$ substrate (characterised by an index of $n_{g}=1.45$ at 1.55 µm [24]). The overlayer is occupied by the volume of the analyte, which has a variable refractive index $n_{a}$. Figure 1b illustrates the unit cell of the MS, which comprises four hollow nanocuboids with cuboid and hole width labelled as *w* and *h*, respectively. The separation between adjacent nanocuboids is denoted as *g*, leading to a pitch value p=2(w+g) for the periodic MS. Figure 1c shows the solid nanocuboid structure that is later studied as a comparative example.

To enable the excitation of the target symmetry-protected quasi-BIC resonant mode, the holes are shifted along the diagonals of the quadrumer, with a relative shift that goes from s=0 (the hole in the nanocuboid centre) to $s_{\max}=\pm 1$ (the hole in the outward or inward corner of the nanocuboid). One advantage of this configuration is that the 2D symmetry of the unit cell (C4v in Schoenflies notation [25]) is maintained for *s* from −1 to 1. Moreover, the First Brillouin Zone (FBZ) is doubled from s=0 to s≠0, aligning the points M, X1, and X2 from the unperturbed FBZ to Γ [26]. This allows the normally incident plane wave to excite the quasi-BIC mode of the MS. In contrast to the conventional techniques, which grant access by decreasing point symmetry, thereby resulting in polarization dependence, this method maintains the unit cell’s (with one cuboid) initial point symmetry C4v. The desired mode access is achieved by modifying the unit cell’s basis and the array’s translational symmetry.

In the case of solid silicon nanocuboids, i.e., for h=0, as shown in Figure 1c, a specific mode results from the coherent interaction within individual elements. Usually, this mode cannot be excited by a normally impinging linearly polarized wave and the resonator cannot emit radiation due to a null electric dipole moment in the *x*–*y* plane. However, a dislocation in nanocuboid positions (s≠0) introduces asymmetry to the field profile, leading to a small electric dipole moment in the resonating field in the *x*–*y* plane. This coincides with the electric field of the incoming wave, enabling coupling with the external radiation and making the mode quasi-BIC and weakly radiative. In the proposed structure, in addition to preserving the initial C4v point symmetry of the unit cell, the field profile asymmetry is considerably lower. This is due to the nanocuboids being static and only the hole acting as an asymmetric factor, which makes for a lower expected dispersion with shifts. In addition, the nanocuboids always have the same gap, maintaining the electric field confinement that is lost in the solid nanocuboid structure when the nanocuboids shift (expected higher Q-factors).

## 3. Results

This section is organised into three subsections devoted to demonstrating the promising performance of the proposed resonant MS. In Section 3.1, the optical response of solid and hollow silicon nanocuboids is calculated while investigating the effect of the shift in the hole and nanocuboid. In addition, an eigenfrequency study is carried out to quantify the dispersion and Q-factor of the resonant mode. Section 3.2 presents the electric field profiles of MS, adequately designed to achieve delocalized electric fields. Finally, Section 3.3 studies two characteristics of the MS that are relevant in various applications, the response to refractive index variations, and the field enhancement in the overlayer.

### 3.1. Robust and High Q-Factor Quasi-BICs

The optical properties of the MS are investigated through the finite element method (COMSOL Multiphysics™) and Rigorous Coupled Wave Analysis (RCWA, RETICOLO [27]) by defining appropriate periodic conditions at the *x*–*z* and *y*–*z* boundary planes of the unit cell. A linearly polarized plane wave impinges perpendicularly on the MS and the power transmittance of the structure is calculated. The results confirm that the incident electromagnetic wave can be arbitrarily polarized, as the design is polarization-independent. Due to the MS array’s subwavelength pitch (p=1 µm) and overlayer index $n_{a}=1.5$, diffraction of the incident beam is zero in the entire investigated spectrum.

**Figure 2:**
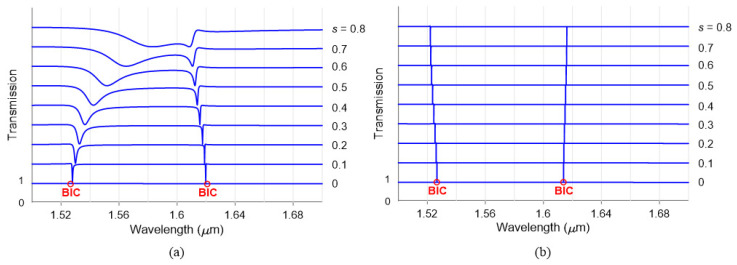
Optical transmittance for different shifts *s* in (**a**) solid nanocuboids and (**b**) hollow nanocuboids (h=50 nm). The nanocuboid has a dimension of w=250 nm, g=250 nm and the surrounding medium has a refractive index of $n_{a}=1.5$.

Figure 2 shows the transmission spectrum for shifts ranging from s=0 (centred) to 0.8 (outwards the diagonal). An RCWA study of the solid and hollow nanocuboids is conducted in Figure 2a and Figure 2b, respectively. Both structures produce two BIC modes, with Mode 2 (manifesting at higher wavelengths) being the sharper one. Another effect is the broadening of the resonance as the shift becomes higher, which is expected for symmetry-protected BICs. One of the problems when using solid nanocuboids to fold the FBZ is that the higher the shift, the higher the gap between nanocuboids. This produces a light leakage that reduces the field confinement. As a result, the resonances are significantly broadened for higher *s* values and start to overlap for high values of *s*, as evidenced in Figure 2a for s=0.8. In the case of hollow nanocuboids, two interesting characteristics arise. The first is a considerably sharper resonance than the solid structure. On the one hand, when the nanocuboids are arranged in an MS configuration, their coupling can lead to very sharp resonances facilitated by their rectangular shape, making such coupling higher than other structures such as nanodiscs. On the other hand, using holes in this kind of structure produces sharper resonances. As theoretically demonstrated in [21] and experimentally in [22], the presence of the central aperture introduces a crucial additional design parameter that governs the field enhancement within the resonant state and determines its spectral location and strength. The second interesting characteristic is the stable resonance wavelength to shift changes. In the case of the solid nanocuboids, the field profile is affected by their movement in the unit cell. In contrast, the hollow nanocuboids maintains a static position with a fixed gap, and the folding of the FBZ is a consequence of the small hole shift.

**Figure 3:**
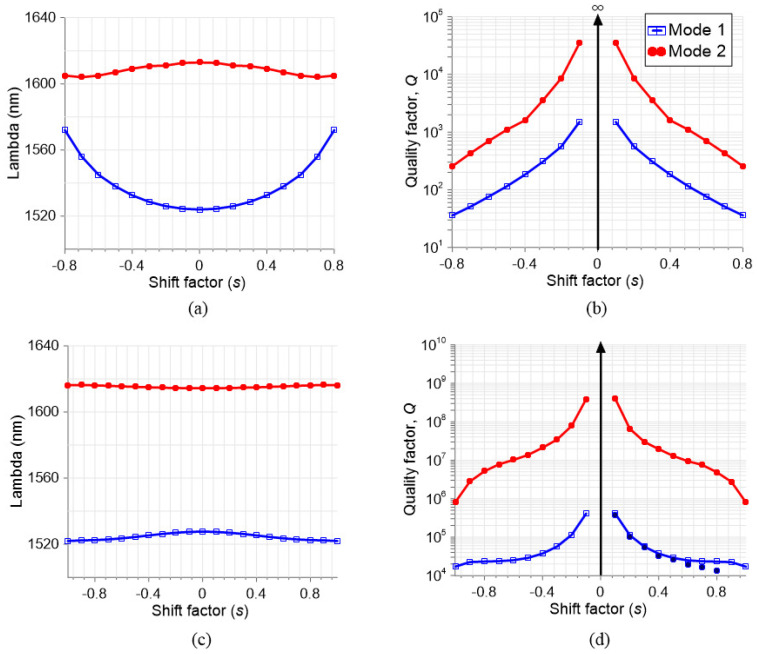
Eigenfrequency study results for different shift factors: (**a**) resonant wavelength for solid nanocuboid, (**b**) Q-factor for solid nanocuboid, (**c**) resonant wavelength for hollow nanocuboid, (**d**) Q-factor for hollow nanocuboid. The solid circles are the RCWA results. The nanocuboid has a dimension of w=250 nm, g=250 nm, the hole h=50 nm, and the surrounding medium has a refractive index of $n_{a}=1.5$.

In Figure 3, an eigenfrequency problem of the structure is solved by COMSOL. The resonance wavelength is plotted in Figure 3a,c. The two eigenmodes of the structure reveal low dispersion for Mode 2 (red) in the solid (a) and hollow (b) nanocuboid structures. Despite this, the dispersion is considerably lower for the latter case. In order to measure the amount of dispersion of the modes with the same units of the curve, the standard deviation σ (the root square of variance) is used. In the case of solid nanocuboids, for Mode 1 σ=15.55 nm and for Mode 2 σ=3.18 nm, whereas for the hollow nanocuboids Mode 1 σ=2 nm and Mode 2 σ=0.7 nm. With respect to the Q-factors in Figure 3b,d, the COMSOL solution is compared to the results in Figure 2 while fitting the calculated transmittance spectra to the Fano function:

(1) $$T\left(\omega_{n}\right)=C\frac{{\left(F\gamma_{n}+\omega_{n}-1\right)}^{2}}{{\left(\omega_{n}-1\right)}^{2}+\gamma_{n}^{2}},$$

where *C* is a scaling parameter, *F* is the Fano parameter that describes the degree of asymmetry, $\omega_{n}=\omega /\omega_{0}$ is the circular frequency normalised to the resonant frequency $\omega_{0}$, and $\gamma_{n}$ is the normalised Fano linewidth. As can be seen in Figure 3d, the results from COMSOL (blue squares) and RCWA (solid blue circles) are fairly in agreement. Here, it is important to highlight the higher Q-factor of the hollow nanocuboid structure with respect to the solid one (almost four orders of magnitude) along with the lower slope, which make for a more stable Q-factor for different hole shifts. In the case of Mode 2, this increase is even greater for both the solid and hollow structures. The best case is observed for Mode 2 in the hollow structure, with Q-factor values that reach $4\times 10^{8}$ for a hole shift of s=0.1 (equivalent to around 10 nm). This shift and the hole size (50 nm) can be obtained with an electron-beam lithography setup, in which sub-10 nm resolution and 30–40 nm feature size are common.

Regarding the losses, it is a well-established fact that quasi-BIC strong resonances are susceptible to the influence of material losses. While polycrystalline silicon exhibits minimal losses within the specified wavelength range, practical considerations such as defects or surface roughness during fabrication can introduce absorption and scattering losses. In a previous work [28], the impact of losses was quantified by introducing the extinction coefficient *k*, which corresponds to the imaginary component of the silicon refractive index. It was observed that when $k<10^{-9}$, a value well within the range of experimental silicon characterisations, the metasurface response remains unaffected for realistically achievable geometries.

### 3.2. Delocalized Electric Field

The control of the electric field profile is a pivotal pursuit in the design of nanostructured surfaces and MS. As mentioned earlier, a promising approach in this field involves Mie resonances in dielectric materials. While Mie resonances exhibit remarkable potential, particular challenges, including light leakage, constrain their optimal confinement and enhancement capabilities. In this context, the current section focuses on an investigation of the delocalized electric field produced in the proposed MS. The MS is designed to produce ultra-high Q-factor delocalized resonances, a marked improvement over prior toroidal responses. Interestingly, the array parameters accentuate the distribution of the electric field predominantly beyond the resonator volume, which could be very important in situations where interactions with outside electric fields are crucial.

**Figure 4:**
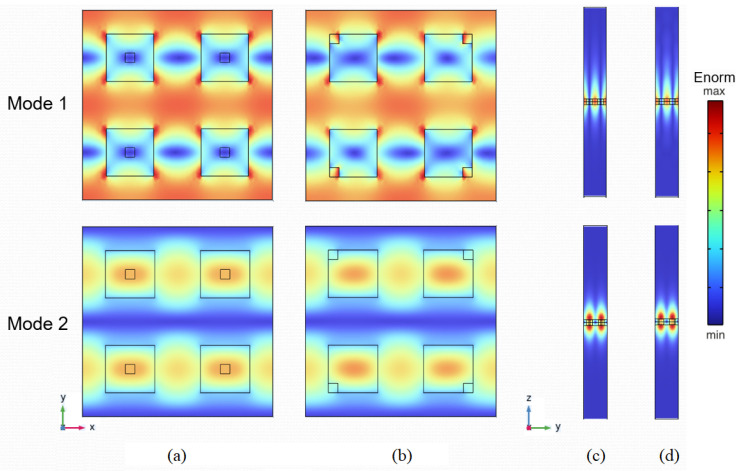
Normalized electric field profile at the *x*–*y* plane: (**a**) s=0, (**b**) s=1 and yz-plane, (**c**) s=0, (**d**) s=1. The nanocuboid has a dimension of w=250 nm, g=250 nm, the hole h=50 nm, and the surrounding medium has a refractive index of $n_{a}=1.5$.

One of the advantages of hole symmetry breaking over the displacement of solid cuboids is that it produces a slight change in the electric field profile and maintains a constant gap. As demonstrated in the previous section, this slight change is enough to generate the quasi-BIC mode and produce a low dispersion for the resonance wavelength. This is corroborated by the electric field profiles of the two BIC (s=0) and quasi-BIC (s=1) resonant modes shown in Figure 4. As can be observed in Figure 4a,b, which corresponds to an x-polarized normally impinging wave, the in-plane profile of the field is only slightly perturbed when the central hole is displaced. In addition, a significant part of the electric field lies outside the silicon resonators, especially in the case of Mode 1. Moreover, another important aspect from the sensing application standpoint is the profile in the upper part of the resonator plane. As can be seen in Figure 4c,d, the electric field spreads hundreds of nanometers over the MS layer for both modes. The absolute values of this enhancement are studied in the next section.

The electric field localization is investigated as a function of the analyte refractive index (overlayer). For this, the integral of the normalized electric field over the total volume is related to the integral over a specific area, e.g., for silicon as in Equation (2) and for the rest of the regions accordingly.

(2) $$E_{\%}=100\cdot \frac{{\;\int \int \int \;}_{V_{\mathrm{Si}}}\left|E\right|dV}{{\;\int \int \int \;}_{V_{\mathrm{tot}}}\left|E\right|dV}$$

**Figure 5:**
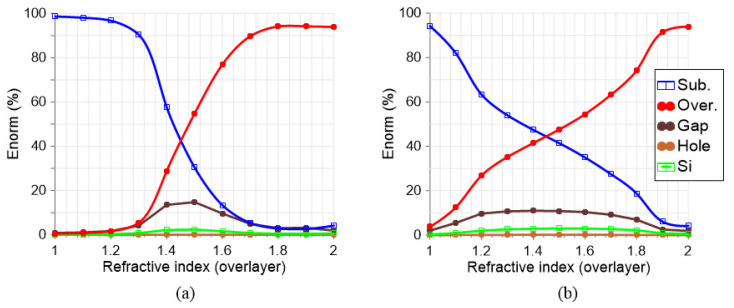
Hollow cuboid structure response, showing the normalized electric field distribution in the different layers of the structure and several refractive indexes of the overlayer: (**a**) Mode 1 and (**b**) Mode 2. The nanocuboid has a dimension of w=250 nm, g=250 nm, the hole h=50 nm, and the shift s=0.1. Legend: Substrate (Sub.), Overlayer (Over.), Gap (volume between nanocuboids), Hole (volume of the hole inside the cuboid), Si (volume of the four cuboids).

As demonstrated in Figure 5a, in the case of Mode 1, the electric field profile in the gap is almost 20% for $n_{a}=1.5$. For a refractive index higher than the substrate (around 1.45), the electric field distribution is mainly in the overlayer, with values reaching 90% for $n_{a}=1.7$. This indicates that high light–matter interaction can be produced in this range. Nevertheless, this mode abruptly loses all of the electric field through the substrate for a refractive index lower than the substrate. On the contrary, Mode 2, investigated in Figure 5b, maintains almost a constant distribution of the electric field at the gap, specifically, 10% from $n_{a}=1.2$ to 1.7. In the overlayer, the electric field ranges from 10% to 60%. This demonstrates robust behaviour even for refractive indexes lower than the substrate, allowing its use in this range.

### 3.3. Optical Sensing and Field Enhancement

**Figure 6:**
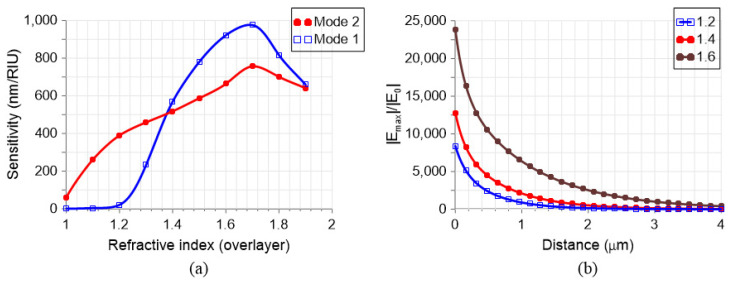
Hollow cuboid structure response: (**a**) Mode 1 and Mode 2 sensitivity for different refractive indices in the overlayer ($n_{a}$) and (**b**) electric field enhancement as a function of the distance from the cuboids (in the overlayer) for three different $n_{a}$. The nanocuboid has a dimension of w=250 nm, g=250 nm, the hole h=50 nm, and the shift s=0.1.

Finally, the most relevant tuning mechanism for sensing applications is the refractive index $n_{a}$ of the surrounding medium. Figure 6a investigates the effect of the surrounding refractive index on the resonant wavelengths for a hollow nanocuboid MS with w=g=250 nm, h=50 nm, and s=0.1. A variation of the refractive index of the overlayer $n_{a}$ shifts the quasi-BIC resonances of the MS. This effect can be used as a sensing mechanism with a sensitivity that depends on $n_{a}$. A broad range of refractive indices has been considered, from $n_{a}=1$ to 2. This range covers all possible analyte materials, such as air and gases (1–1.1), water, biosolutions (1.3–1.5), and various optical liquids (1.5–2).

As expected, following the results of Figure 5a, the sensitivity for Mode 1 drops abruptly for a refractive index lower than that of the substrate. Nevertheless, for a refractive index of $n_{a}=1.7$, a sensitivity $S=\Delta \lambda_{r}/\Delta n_{a}$ equal to 1000 nm/RIU is obtained. This is a very high value compared to other dielectric structures (with sensitivities around 100–700 nm/RIU; see Table 1 in [18]) and even certain plasmonic counterparts (e.g., based on LSPR and SLR). While Mode 2 reaches values up to 800 nm/RIU, it has a lower decrease when the refractive index is lower than the substrate, with a value of 460 nm/RIU for $n_{a}=1.33$ (water). The sensitivity drops to 60 nm/RIU for air, indicating that it remains usable at this minimum value. It has to be considered that Q-factors for Mode 2 range from around $10^{8}$ to $10^{9}$ for $n_{a}$, ranging from 1.3 to 1.6, respectively. This very sharp resonance feature produces a very high figure of merit (FoM), namely, the sensitivity divided by the FWHM (full width at half maximum).

Another important feature in applications requiring light–matter interaction (such as spectroscopy, SERS, etc.) is the enhancement of the electric field in the overlayer. In Figure 6b, the maximum electric field enhancement of Mode 2 in the *x*–*y* plane at a specific distance above the nanocuboids is calculated. For this, the maximum electric field in this plane is divided by the incident electric field (${\left|E\right|}_{\max}/\left|E_{0}\right|$). Three different refractive indices are considered; the higher the refractive index, the higher the enhancement, as there is more field in the overlayer. There is an exponential decrease as the plane moves farther away from the resonator, as expected; for example, considering $n_{a}=1.4$, a close distance of 100 nm corresponds to an enhancement of 10,000, whereas a 2000 enhancement factor is produced for 1 µm of separation.

## 4. Conclusions

In this work, we have demonstrated a novel structure based on a hollow nanocuboid cluster MS that supports quasi-BIC modes. The proposed hole symmetry-breaking mechanism has several advantages: normal coupling, polarization independence, an ultra-high radiative Q-factor, and delocalized field confinement. Furthermore, this MS paves the way for different applications in which light–matter interaction is critical, e.g., refractometric sensing and spectroscopy. In this regard, while other methods demonstrate sensitivities surpassing the proposed refractometric sensor (e.g., SPR), the latter possesses distinct advantages typical of high-Q dielectric metasurfaces [29], showcasing its potential: (i) it boasts a sub-picometric linewidth, which, when coupled with cutting-edge optical spectrum analysers and temperature stabilization modules, can substantially lower the detection limit, ultimately constrained by the analyte absorption properties; (ii) it maintains compact dimensions, is limited by the probe beam’s size, features a planar design involving a single lithographic step, and functions in free space; (iii) the design is scalable to other target wavelengths through simple geometric adjustments thanks to the low material dispersion of silicon; (iv) the configuration is all-dielectric, sidestepping ohmic losses encountered in plasmonics-based sensors; (v) it holds the potential for integration into microfluidic setups, which is achieved by containing the analyte volume with a planar overlayer; and (vi) ultimately, by reversing its operational principle, the same device could serve as a remarkably selective tunable optical notch filter. This would involve substituting the analyte material with an electro-optically tunable medium such as nematic liquid crystals. Moreover, scaling of the proposed structure is in principle possible towards other spectrum ranges (e.g., visible) for advanced spectroscopy by using suitable transparent materials (e.g., TiO${}_{2}$ or GaP).

## Data Availability

The datasets generated during and/or analyzed during the current study are available from the corresponding author on reasonable request.
